# Mitochondrial quality-control uncoupling in sepsis: a missing link to persistent immune hyporesponsiveness

**DOI:** 10.3389/fimmu.2026.1982407

**Published:** 2026-10-20

**Authors:** Yu Zhang, Xin Ping Xia, Ling Zhao

**Affiliations:** 1 Department of Rehabilitation Medicine, The People’s Hospital Medical Group of Xiangzhou, Zhuhai, Guangdong, China; 2 Department of Intensive Care Medicine, The People’s Hospital Medical Group of Xiangzhou, Zhuhai, Guangdong, China

**Keywords:** immune hyporesponsiveness, immunometabolism, lysosome, mitochondrial biogenesis, mitochondrial quality control, mitophagy, sepsis

## Abstract

Sepsis imposes competing demands for antimicrobial activity and organelle maintenance. Mitochondrial recovery requires coordinated damage containment, selective removal, lysosomal degradation and recycling, and functional replacement. We define mitochondrial quality-control uncoupling as a mismatch among these processes. When damage outpaces completed clearance, dysfunctional mitochondria retain redox and danger-signaling activity. When removal outpaces replacement, mitochondrial mass and spare respiratory capacity decline. Incomplete lysosomal processing can combine both phenotypes by removing mitochondria from the functional network while retaining undegraded cargo. Immune cells may therefore remain viable yet fail to increase metabolic and biosynthetic output during renewed challenge. Inhibitory immune signaling can further restrict substrate use and mitochondrial rebuilding, reinforcing mitochondrial and immune dysfunction. This framework reconciles divergent observations on mitophagy and explains how inflammation can coexist with ineffective host defense. We define state-matched longitudinal phenotyping as repeated, cell-type-specific assessment of damage burden, clearance completion, respiratory reserve and immune function over time. Such phenotyping could guide interventions toward the dominant defect rather than indiscriminately activating a single pathway.

## 1 Introduction

Sepsis is clinically defined as life-threatening organ dysfunction caused by a dysregulated host response to infection (1). This definition captures the severity of the syndrome, but it does not explain why patients with similar hemodynamic disturbances and organ-failure scores show markedly different biological courses. Some patients recover after a relatively transient period of metabolic and immune suppression, whereas others develop persistent defects in antigen presentation and antimicrobial defense that are associated with increased susceptibility to secondary infections (2). Persistent inflammatory activity may also coexist with immune hyporesponsiveness, indicating that hyperinflammation and immune failure are not simply consecutive phases but can overlap and evolve along distinct biological trajectories (3).

Mitochondria may help connect these seemingly contradictory features. During infection, immune cells must support migration, phagocytosis, membrane trafficking, cytokine production, clonal proliferation, immune-synapse formation and cytotoxic activity. These functions require extensive metabolic reprogramming involving glycolysis, oxidative phosphorylation, lipid and amino-acid metabolism, together with the generation of biosynthetic intermediates rather than basal ATP production alone (4). Mitochondria also regulate redox signaling and provide metabolic platforms for immune responses. When mitochondrial homeostasis is disrupted, mitochondrial reactive oxygen species (mtROS), mitochondria-derived nucleic acids, including mitochondrial DNA (mtDNA), lipids and other mitochondrial damage-associated molecular patterns (mtDAMPs) may sustain inflammatory pathways while impaired mitochondrial bioenergetics constrains effective host defense (5).

Previous studies have often addressed mitochondrial dysfunction and immune hyporesponsiveness in sepsis as partly separate biological problems. Mitochondrial studies have focused on respiratory-chain dysfunction, loss of membrane potential, oxidative stress, mtDNA release and impaired cellular or tissue bioenergetics (6, 7). In parallel, immunological studies have emphasized lymphocytopenia, monocyte deactivation, impaired recall responses, reduced natural killer-cell function, increased expression of inhibitory receptors, and exhaustion-like phenotypes (8, 9). A simple model in which mitochondrial injury reduces ATP availability and thereby suppresses immune function is useful but incomplete. It does not explain why mitophagy can be protective in some settings yet become insufficient or potentially maladaptive when clearance and mitochondrial replacement are not appropriately coordinated (6), or why mitochondrial danger signaling may persist despite impaired antigen presentation and antimicrobial immune responses (7, 9).

The missing link may lie in the maintenance system that determines the fate of damaged mitochondria. Under physiological conditions, mitochondrial quality control integrates damage containment, selective removal of irreversibly injured mitochondrial components, lysosomal processing, recycling, and restoration of functional mitochondrial capacity through repair and biogenesis (10). These processes are functionally interdependent: selective mitochondrial removal must be balanced by mitochondrial biogenesis and restoration of the functional mitochondrial pool to maintain cellular homeostasis (11). We define the loss of this coordination as mitochondrial quality-control uncoupling.

This idea helps resolve a central contradiction in sepsis biology. When mitochondrial damage exceeds completed clearance, dysfunctional mitochondria may accumulate and continue to generate reactive oxygen species and mitochondrial danger signals (5). Conversely, when mitochondrial loss is not adequately matched by restoration of functional mitochondrial capacity, mitochondrial mass and respiratory reserve may decline (12). Moreover, initiation of mitophagy does not necessarily indicate successful mitochondrial clearance; if downstream lysosomal processing is incomplete, mitochondrial cargo may enter the degradative pathway without being fully eliminated (6). These situations are not necessarily contradictory; within the framework proposed here, they can be interpreted as different rate mismatches within the same mitochondrial maintenance process.

Quality-control uncoupling may provide a mechanistic link between organelle damage and persistent immune hyporesponsiveness. Accumulated dysfunctional mitochondria can perturb redox and innate immune signaling, whereas loss of functional mitochondrial capacity can reduce the metabolic reserve required for high-demand immune functions (7, 12). Consequently, immune cells may remain viable under basal conditions yet fail to mount an adequate response when metabolic demand rises during re-stimulation (13). In parallel, persistent inhibitory immune programs can restrict PI3K–AKT–mTOR-dependent metabolic reprogramming, glucose utilization, anabolic metabolism, and mitochondrial fitness, potentially creating a reciprocal constraint on immune and mitochondrial recovery (14). Thus, we propose that an initially reversible stress response may become self-reinforcing: impaired mitochondrial maintenance weakens immune function, while immune inhibition further constrains mitochondrial recovery. The likelihood of such a transition may also depend on baseline physiological reserve. Aging, frailty, comorbidities, and pre-existing immunosuppression are associated with diminished physiological and immune resilience and may increase vulnerability to persistent dysfunction after septic stress (15, 16). In this paper, we therefore consider mitochondrial quality-control uncoupling as an organizing principle for persistent immune hyporesponsiveness in sepsis and explore how this framework might inform state-specific phenotyping and intervention. The operational definition used throughout this review is summarized in **Box 1**.

Box 1 **Operational definition of mitochondrial quality-control uncoupling.**

**Table:** 

Component	Operational meaning
Damage load	Amount of mitochondrial damage production and the quantity of newly damaged organelles requiring repair or removal (10).
Selective removal	Preferential removal of irreversibly damaged mitochondria while limiting the loss of recoverable mitochondrial capacity (17).
Lysosomal completion and recycling	Fusion with lysosomes, degradation of cargo, and return of reusable products to the cytosol (18).
Functional replacement	Restoration of competent respiratory capacity by means of repair, protein import, fusion, mitochondrial DNA conservation and biogenesis (19).
Uncoupling	Rate mismatches among these processes produce accumulation, depletion, lysosomal congestion or a mixed state.

This box proposes a conceptual framework for mitochondrial quality-control uncoupling in sepsis. (The abbreviations are given in the Abbreviations part).

## 2 Literature search strategy

We carried out a structured narrative search for the mechanistic and translational evidence on the relationship between sepsis, mitochondrial maintenance and immune hyporesponsiveness. The studies published from 1 August 2021 to 31 July 2026 were searched in PubMed/MEDLINE, Web of Science Core Collection and Scopus. Pre-2021 studies were included if they described fundamental mechanisms, such as PINK1-Parkin-mediated mitophagy, receptor-mediated mitophagy, autophagosome-lysosome flux, mitochondrial biogenesis and inhibitory immune-metabolic signaling. Human sepsis studies were given priority for clinical interpretation while animal and cell-based studies were used when they explained the causal steps that cannot be measured directly in patients. Original studies were used to support specific mechanistic claims; high-quality reviews and consensus documents supplied the broader context. The detailed PubMed/MEDLINE strategy is given at the end of the paper.

We also performed a targeted update for experimental sepsis studies that measured mitophagy with pH-sensitive reporters or paired pathway markers with mitochondrial mass, respiratory function, lysosomal function or biogenesis. We prioritized macrophages, monocytes and other inflammatory cells, while retaining organ studies when they directly resolved the balance between mitochondrial removal and replacement. This evidence was used to distinguish pH-sensitive reporter-based measurements of mitochondrial delivery to acidic lysosomal compartments from static measurements of pathway components and to evaluate the extent to which the observations were consistent with each of the three proposed states.

Additional searches addressed mechanistic relationships not fully captured by the main strategy, including mitochondrial protein import, mitophagic flux, lysosomal completion and recycling, respiratory reserve, immune-cell recall and inhibitory immunometabolic signaling. We also examined the reference lists and forward citations of key papers. Studies were selected when they clarified a specific stage linking mitochondrial injury, impaired maintenance, functional replacement, immune hyporesponsiveness or reverse inhibitory feedback. Because models, cell types, assays and outcomes were heterogeneous, we synthesized the evidence narratively rather than quantitatively.

## 3 The mitochondrial maintenance chain

Mitochondrial quality control is often reduced to mitophagy, although recovery depends on a sequence of linked molecular transitions (20, 21). Fission first segregates damaged domains, selective tagging then commits irreversibly injured material to mitophagy, and lysosomal processing converts disposal into reusable substrates. Recycling, protein import and biogenesis subsequently restore respiratory capacity and reintegrate mitochondria into the functional network (22). Failure at one checkpoint changes the burden on the next and can feed back onto earlier steps (23). The following subsections trace these transitions from damage segregation to functional replacement. The mitochondrial maintenance chain and its potential links to immune hyporesponsiveness in sepsis are illustrated in **Figure 1**.

**Figure 1:**
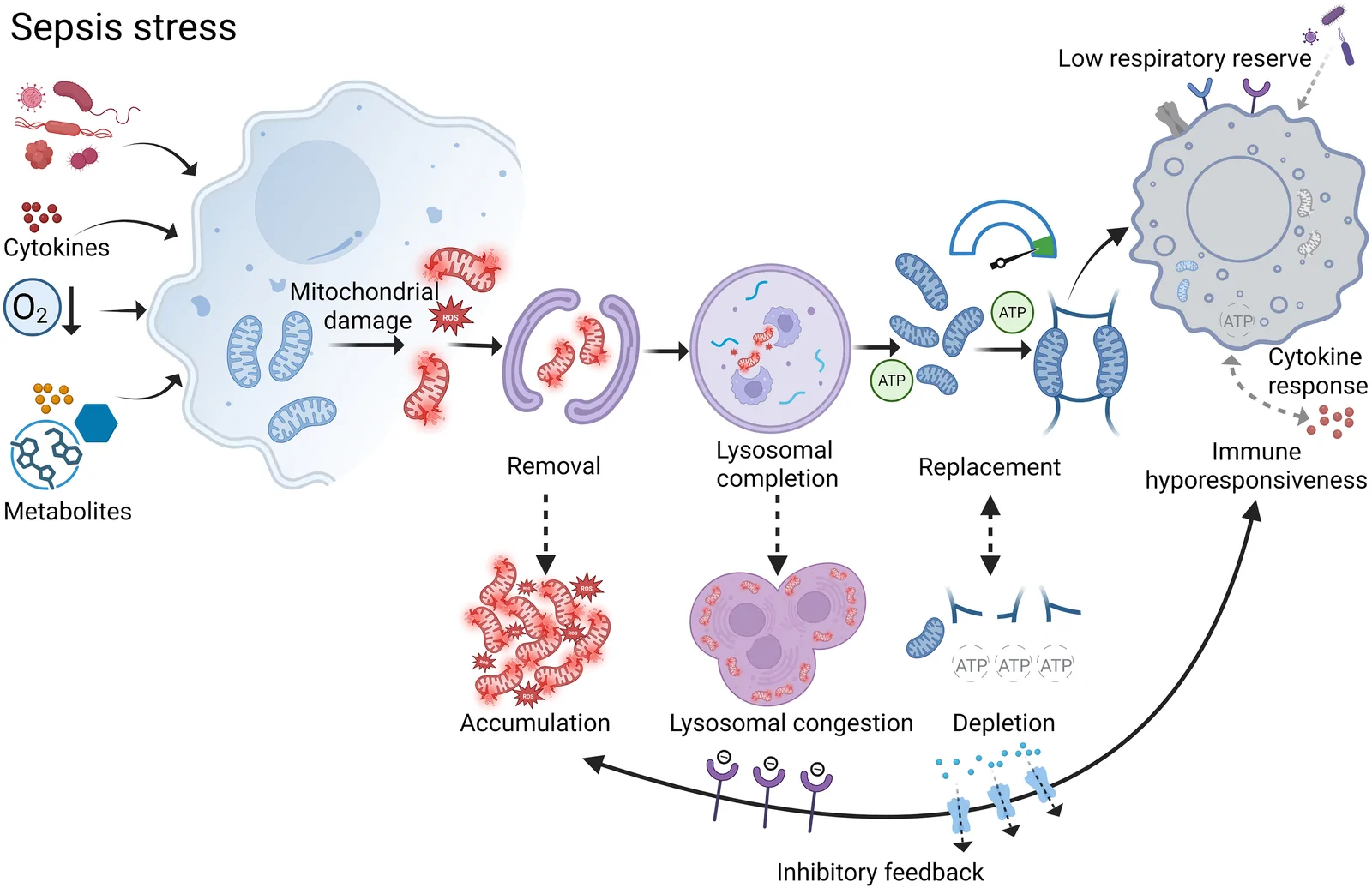
Mitochondrial quality-control failure and immune hyporesponsiveness in sepsis. Sepsis-related inflammatory, hypoxic, and metabolic stress induces mitochondrial damage, which is normally contained through mitochondrial removal, lysosomal degradation, and functional replacement. Failure at these successive steps produces three dominant patterns: damaged-mitochondrial accumulation, lysosomal congestion, and mitochondrial depletion with reduced ATP and respiratory reserve. These defects impair the ability of immune cells to meet the metabolic demands of a subsequent challenge, contributing to immune hyporesponsiveness. Inhibitory signaling and reduced nutrient uptake may further reinforce this dysfunctional state and hinder mitochondrial recovery. Created in BioRender. Zhang, Y. (2026) https://https://BioRender.com/lw7kqqo (The abbreviations are given in the Abbreviations part).

### 3.1 Containing local damage

Mitochondrial networks continuously undergo fission and fusion (24). Fission can separate a severely depolarized or oxidized segment from healthier neighbors, thereby limiting the spread of local damage (25). Fusion allows partially injured mitochondria to exchange metabolites, proteins and mitochondrial gene products, preserving function when repair remains possible. Neither process is intrinsically beneficial or harmful (23). The outcome depends on whether fission selectively isolates irreversibly damaged domains while the remaining network retains sufficient fusion and respiratory capacity.

During early stress, fission can partition damaged regions into removable units (25). Persistent stress can disrupt this balance. Repeated fission may generate more fragments than downstream pathways can process, while fusion no longer compensates for ongoing loss (26). As the clearance burden rises, recovery becomes increasingly dependent on autophagic and lysosomal capacity (27). Fission therefore contains the initial lesion but does not complete repair. Selective tagging must next direct damaged fragments toward removal without depleting the entire network (11, 28).

### 3.2 Selective removal

Loss of mitochondrial membrane potential stabilizes PINK1 on the outer membrane and promotes Parkin-dependent ubiquitination (29). Receptor-mediated pathways can also connect damaged mitochondria directly to LC3-family proteins without Parkin (30, 31). The major mitophagy-recognition routes and their activation logic are summarised in **Box 2**. These pathways distinguish organelles committed to elimination from those that can still support cellular reserve (22). This selectivity is particularly important because immune cells may operate near their energetic limit during sepsis (32). Removing an irreversibly damaged mitochondrion reduces oxidative stress and danger signaling. Removing a partially functional mitochondrion, however, may worsen energetic failure if replacement is delayed (11).

Box 2 **Major mitophagy-recognition routes and activation logic.**

Mitophagy can be initiated through ubiquitin-dependent recognition or by mitochondrial receptors. Major routes characterized primarily in mammalian cell systems are summarized below.

**Table:** 

Route	Principal activation logic
PINK1–PRKN ubiquitin pathway	Loss of membrane potential stabilizes PINK1 on the outer mitochondrial membrane (33). PINK1 phosphorylates ubiquitin and activates PRKN (34), after which OPTN and CALCOCO2/NDP52 recruit the autophagy machinery to ubiquitinated mitochondrial cargo (35).
BNIP3 and BNIP3L/NIX	BNIP3 and BNIP3L/NIX bind LC3/GABARAP-family proteins through LC3-interacting regions. Phosphorylation adjacent to their LIR motifs strengthens LC3-family binding and mitochondrial sequestration (36, 37), while BNIP3/NIX-dependent LC3 engagement supports tight isolation-membrane attachment and expansion (31).
FUNDC1	FUNDC1 mediates hypoxia-induced receptor mitophagy and is regulated by phosphorylation. Hypoxia-associated dephosphorylation promotes LC3 binding (38), whereas ULK1-mediated phosphorylation at Ser17 further increases FUNDC1–LC3 engagement (39).
BCL2L13 and FKBP8	BCL2L13 binds LC3 through an LIR and can induce PRKN-independent mitophagy (40). FKBP8 recruits LC3A to damaged mitochondria and supports PRKN-independent mitophagy (41).
PHB2	PHB2 is an inner mitochondrial membrane receptor exposed after PRKN/proteasome-dependent outer-membrane rupture; its LIR then binds LC3 (42).

The table summarizes receptor pathways characterized primarily in mammalian cultured-cell systems. Inclusion does not imply direct validation in septic immune cells.

Mitophagy initiation alone does not establish successful quality control. Increased PINK1, Parkin recruitment, LC3 lipidation, receptor expression or mitochondrial fragmentation indicates pathway entry, not lysosomal delivery, cargo degradation or restored function (18, 22). Interpretation therefore requires staged assessment of mitolysosome formation, cargo degradation and functional recovery rather than static markers alone. Autophagosome sequestration marks the transition from selective tagging to lysosomal processing (43).

### 3.3 Lysosomal completion and recycling

When an autophagosome encloses a mitochondrion, the organelle has left the functional network but has not yet been cleared (10). The autophagosome must fuse with an acidic, enzyme-competent lysosome (44). Lysosomal hydrolases then degrade mitochondrial proteins, lipids and nucleic acids, while membrane transporters return recyclable amino acids, fatty acids, nucleotides, iron and other components to the cytosol (27). This transition converts disposal into recovery. Without it, the cell loses a functional organelle without eliminating the injurious cargo or recovering substrates for repair (10).

Lysosomal completion depends on ATP, proton gradients, membrane trafficking, fusion machinery, active hydrolases and intact lysosomal membranes (45). In sepsis, acidosis, hypoxia, oxidative damage, inflammatory responses and ATP depletion may interfere with these processes (44, 46). Damage recognition, mitophagosome formation and vesicular transport may continue even when terminal processing slows. Failed fusion causes mitochondria-containing autophagosomes to accumulate before lysosomal entry. If fusion occurs but acidification, hydrolysis or substrate export fails, cargo reaches autolysosomes without complete processing (27). Strong mitophagy signals can therefore coexist with residual undigested material, separating pathway initiation from lysosomal completion.

### 3.4 Functional replacement

Complete recovery requires restoration of the functional mitochondrial capacity that was lost (21). Biogenesis, repair, fusion, protein import, mtDNA maintenance and network integration contribute to this process (47). These steps require coordinated nuclear and mitochondrial transcription, respiratory-protein and membrane-lipid synthesis, mtDNA replication, membrane-potential-dependent protein import and network incorporation. Increased PGC-1α, nuclear respiratory factors or TFAM may indicate compensation but do not establish restored respiration (48).

Functional replacement may fail even when biogenesis pathways are activated. Loss of membrane potential impairs the import of nuclear-encoded mitochondrial proteins (49). Oxidative stress damages newly synthesized components (21). Ineffective lysosomal degradation restricts substrates for membrane and respiratory-complex formation, while persistent inflammatory signals suppress repair programs (47, 50). Consequently, the cell may activate biogenesis yet retain low maximal respiration and spare respiratory capacity. Functional recovery should therefore be judged by restored performance rather than a single biogenesis marker (48).

## 4 How septic stress overwhelms mitochondrial maintenance

Septic stress both damages mitochondria and weakens the systems required for repair (51). Pattern-recognition signaling and cytokines increase the production of reactive oxygen and nitrogen species (52); microcirculatory disturbance alters the supply of oxygen and substrates (53); calcium balance becomes unstable (53); and circulating nutrients may remain abundant while intracellular utilization declines. Each disturbance may be tolerated for a short period, but together they increase damage burden while reducing recovery capacity (3, 51).

### 4.1 Electron-transport injury creates an oxidative amplification loop

The electron transport chain is a major site at which oxidative and nitrosative modifications impede electron transfer, over-reduce carriers and promote electron leakage to molecular oxygen, particularly at complexes I and III (54, 55). The resulting mtROS oxidize respiratory proteins, mtDNA and cardiolipin (56). The peroxidation of cardiolipin disrupts the inner membrane and respiratory supercomplexes, which further impedes electron flow and increases leakage (57).

This feedback can convert a local lesion into network-wide injury. Inefficient electron transfer increases mitochondrial oxidants, which further damage the electron-transfer machinery (55). Glutathione, NADPH and repair systems buffer this cycle while reserve remains adequate (58). Once injury exceeds buffering and repair capacity, mtROS become structural stressors and expand the mitochondrial population requiring repair or elimination (56).

Peroxynitrite injures iron-sulfur centers and respiratory-chain proteins, and cardiolipin peroxidation disturbs cristae and respiratory supercomplexes (59, 60). mtDNA damage also reduces synthesis of mitochondrially encoded respiratory subunits. Together, these lesions can convert a transient oxidative burst into persistent loss of electron-transfer efficiency and sustained demand for repair or removal (58).

### 4.2 Membrane-potential loss links energy failure to repair failure

Mitochondrial calcium uptake can support energy production by activating mitochondrial dehydrogenases (61). Nevertheless, prolonged calcium exposure results in permeability transition, swelling, cristae disruption and loss of membrane potential (62). This decline in potential decreases ATP synthesis and impairs mitochondrial self-maintenance (63). Because most mitochondrial proteins are nuclear encoded and imported in a membrane-potential-dependent manner, depolarized mitochondria cannot efficiently replace defective proteins, maintain mtDNA or restore respiratory complexes (64).

Loss of membrane potential compromises both bioenergetic output and repair. ATP depletion slows vesicle transport, autophagosome maturation, lysosomal acidification and substrate export (65, 66). Depolarization also impairs protein import, preventing replacement of damaged respiratory components. Electron transfer remains inefficient, oxidant production persists and membrane potential declines further (55). Compensatory transcription alone therefore cannot complete clearance or restore mitochondrial function.

### 4.3 Metabolic availability becomes disconnected from metabolic use

Sepsis does not simply deplete circulating nutrients; it disrupts the conversion of extracellular nutrients into usable intracellular carbon (67). Activated myeloid cells may initially accelerate glycolysis, and lymphocytes increase glucose uptake, glycolysis and mitochondrial oxidation after receptor stimulation (68). Under sustained stress, cells may fail to induce glucose transporters, oxidative modifications may inhibit glycolytic enzymes, and reduced pyruvate dehydrogenase activity may restrict pyruvate conversion to acetyl-CoA (67). Plasma glucose may therefore remain high while insufficient carbon enters the tricarboxylic acid cycle, oxidative phosphorylation and biosynthetic pathways (3).

Lipids and amino acids show a similar mismatch between availability and utilization. Stress releases fatty acids, but impaired mitochondrial uptake, reduced oxidative programs and respiratory-chain defects limit complete oxidation, causing lipid intermediates to accumulate and increasing membrane and redox stress (67). Muscle proteolysis releases amino acids, yet gluconeogenesis, acute-phase synthesis, antioxidant production, immune responses and tissue repair consume them rapidly. Some cells may therefore lack anaplerotic and biosynthetic substrates despite apparently sufficient circulating concentrations (69).

These defects affect the mitochondrial maintenance system. Limited carbon entry reduces ATP production and redox regeneration; insufficient NADPH and glutathione weaken antioxidant defense (68); and the deficiency of amino acids and lipids hinders the synthesis of respiratory proteins and mitochondrial membranes (4). Autophagy may be initiated while lysosomal completion and functional replacement lag. As usable fuel, repair energy and biosynthetic substrates decline, damaged material accumulates or mitochondria are removed without replacement, further reducing respiratory capacity. Thus, high blood glucose and abundant circulating substrates can coexist with intracellular energetic and biosynthetic insufficiency because circulating nutrients do not necessarily become available within immune cells (3).

## 5 Three forms of mitochondrial quality-control uncoupling

Mitochondrial quality-control uncoupling occurs when the processes of damage generation, selective removal, lysosomal completion and functional replacement proceed at incompatible rates (11). Three archetypal states capture the principal consequences: accumulation-dominant uncoupling, depletion-dominant uncoupling and a lysosomal-congested mixed state. Baseline reserve, cell type and disease phase determine which state predominates. A cell population may transition between states or display combined features over time (70). The major uncoupling patterns, their mitochondrial and immune consequences, and the corresponding mechanistic priorities are summarised in **Table 1**.

**Table 1:** Major patterns of mitochondrial quality-control uncoupling and their implications.

Pattern	Core imbalance	Mitochondrial phenotype	Immune consequence	Predicted mechanistic priority
Accumulation-dominant uncoupling	New injury outpaces selective removal and terminal processing	Damaged mitochondria, mtROS, and mtDAMPs persist	Inflammatory signaling persists while recall and specialized functions weaken (2, 7)	Reduce ongoing damage and restore completed clearance
Depletion-dominant uncoupling	Mitophagic loss outpaces repair, fusion, and functional replacement	Mitochondrial mass and spare respiratory capacity decline	Cells survive at rest but fail during renewed high-demand work (12, 100)	Protect residual mitochondria and restore functional replacement
Lysosomal-congested mixed state	Cargo delivery outpaces fusion, degradation, or substrate export	Low functional mass coexists with retained damaged cargo and lysosomal congestion	Energy-producing capacity, signaling fidelity, and intracellular substrate availability decline (18)	Restore terminal lysosomal processing before increasing cargo delivery
Re-coupled recovery	Damage, removal, recycling, and replacement regain compatible rates	Mitochondrial quality, mass, and reserve recover	Antigen presentation, recall, proliferation, and cytotoxicity improve (10)	Maintain infection control and support coordinated recovery

The proposed patterns are conceptual states intended to link dominant mitochondrial quality-control failures with their likely immune consequences and mechanistic priorities. (The abbreviations are given in the Abbreviations part.).

### 5.1 Accumulation-dominant uncoupling: damage exceeds completed clearance

Accumulation-dominant uncoupling occurs when new mitochondrial damage exceeds the capacity of the clearance pathway. If recognition, autophagosome formation, lysosomal fusion, acidification, proteolysis or substrate export cannot keep pace, dysfunctional mitochondria and partially digested material accumulate (71).

Retained organelles can continue to exert metabolic and immunological effects. Impaired electron transport promotes the continuous production of mtROS (72), and mtDNA, oxidized cardiolipin, formylated peptides and other mtDAMPs continue to activate innate receptors (73). Persistent redox stress also perturbs kinases, phosphatases, receptors, membrane lipids and metabolic enzymes, reducing the fidelity of immune signaling (74). The cell is therefore not globally inactive. Selected inflammatory pathways may remain active while coordinated antimicrobial functions weaken (75).

pH-sensitive mitophagy reporters detect mitochondrial delivery to acidic lysosomal compartments. mt-Keima undergoes a pH-dependent excitation shift after lysosomal delivery, whereas the tandem mCherry–GFP mito-QC reporter produces mCherry-only puncta because lysosomal acidity quenches GFP while mCherry fluorescence persists (76, 77). These reporters measure mitolysosome formation more directly than static pathway markers, although they do not by themselves establish complete cargo degradation or substrate recycling.

An accumulation-like pattern can occur without increased pathway-entry markers. In macrophages, LPS/IFN-γ inhibited PINK1-dependent mitophagy measured with mt-Keima while mitochondrial density and mtROS increased; CLP and septic-patient monocytes showed a related suppression of mitophagy (76). Complementary loss-of-function evidence in bone-marrow-derived macrophages showed that APPL1 deletion likewise impaired mitophagy, leading to damaged-mitochondrial accumulation, oxidized cytosolic mtDNA and enhanced NLRP3 activation; hematopoietic APPL1 deficiency also increased susceptibility to endotoxin-induced sepsis (78). Together, these observations link impaired mitophagy to damaged-mitochondrial accumulation and inflammatory activation. They also show why successful quality control cannot be inferred from pathway-entry markers or mitolysosome formation alone without concurrent measurements of cargo degradation, lysosomal capacity, mitochondrial mass and respiratory function.

Completed clearance is a multistep process rather than a single molecular signal. It requires recognition, sequestration, transport, fusion, acidification, degradation and release of recyclable material (45). A distal defect may increase upstream markers because cargo enters faster than it exits. Static evidence of pathway activation therefore indicates attempted clearance, whereas time-resolved reporter measurements combined with lysosomal-function and cargo-turnover assays are needed to demonstrate elimination of oxidant-producing mitochondria (18, 43).

### 5.2 Depletion-dominant uncoupling: removal exceeds functional replacement

Depletion-dominant uncoupling cannot be inferred from fragmentation alone. Fragmentation can segregate damaged domains, but commitment to mitophagy requires selective tagging or receptor engagement. In experimental sepsis, depolarization activated CaMKI before PINK1/PRKN and DJ-1 recruitment in macrophages and hepatocytes, showing that stress sensing and signaling, rather than morphology alone, govern pathway entry (79). Assigning a depletion-dominant state therefore requires evidence that selective mitochondrial removal exceeds repair and biogenesis, together with a net loss of mitochondrial mass or respiratory capacity that is not explained by cell death or dilution.

Transient mitochondrial loss has been observed in experimental sepsis, but it does not necessarily establish persistent depletion-dominant uncoupling. In CLP liver and LPS-treated hepatocytes, mitochondrial density and number fell early as autophagy and mitophagy increased, then recovered with induction of mitochondrial biogenesis (80). A similar recoupling pattern was observed in LPS-exposed THP-1 cells and primary human monocytes, where endotoxin tolerance coincided with chloroquine-sensitive LC3 turnover, induction of mitochondrial biogenesis and antioxidant defenses, and recovery of mtDNA, TFAM, mitochondrial mass and respiration (81). Together, these trajectories support transient depletion followed by recoupling rather than persistent unopposed clearance. Direct evidence that mitochondrial removal persistently exceeds replacement in immune cells remains limited.

The key variable is not mitophagy activity alone, but whether functional replacement matches mitochondrial removal (22). Retaining damaged organelles sustains oxidative stress and danger signaling, whereas excessive removal leaves insufficient respiratory machinery for renewed demand (26, 82). Mitophagy is beneficial only when lysosomes can complete degradation and the cell can replace the removed capacity.

Functional impairment depends on workload. A resting cell may tolerate substantial mitochondrial loss because housekeeping functions require limited output. The same cell may fail during antigen-driven proliferation, phagocytosis, membrane trafficking or tissue repair, when ATP and biosynthetic demand rise sharply (83). Residual respiratory reserve therefore buffers mitochondrial loss (32). Once that reserve is exhausted, even modest further loss can markedly impair immune function without compromising cell viability (84).

### 5.3 Lysosomal-congested mixed state: completion and recycling fail

A lysosomal-congested mixed state arises when damaged mitochondria enter the autophagy–lysosome pathway but terminal processing is impaired. Direct evidence specific to mitochondrial cargo in septic immune cells remains limited. Post-sepsis macrophage studies nevertheless identify a related defect in shared clearance machinery: Parkin-deficient macrophages failed to form phagolysosomes and kill bacteria, and Parkin abundance was reduced in leukocytes from sepsis survivors and in post-CLP mice (85). These findings link impaired autophagic and phagolysosomal handling to immune dysfunction but do not directly demonstrate that mitochondrial cargo congests lysosomes.

A terminal bottleneck removes mitochondria from the functional network while damaged cargo or mitophagosomes persist. In macrophages, SESN2 loss reduced LPS/ATP-induced mitophagy and increased inflammasome activation and mortality in CLP and endotoxemia (86). Consistent with distal failure, PICK1-deficient CLP mice and bone-marrow-derived macrophages accumulated autophagosomes, formed fewer autolysosomes and expressed less LAMP1 and cathepsin B (87). However, neither study measured lysosomal occupancy or cargo export, and the PICK1 study did not identify retained cargo as mitochondrial; they therefore support failed autophagic completion, not mitochondrial-cargo-specific congestion.

Conceptually, lysosomal congestion could explain how reduced mitochondrial mass coexists with damaged organelles and why increased mitophagy initiation may worsen dysfunction when lysosomal completion is limiting (43). Additional cargo delivery into a terminally constrained pathway removes organelles from use without correcting the downstream defect.

Several characteristics of sepsis can lead to this obstruction. Acidosis and ATP depletion affect proton pumping and lysosomal acidification (88); oxidative damage alters hydrolases and membrane proteins (89); calcium and transport abnormalities interfere with the fusion of autophagosomes and lysosomes (90); and continuous cargo supply may surpass the renewal of lysosomes regulated by TFEB (91, 92). These demands converge on a shared lysosomal pool. Mitophagosomes and phagosomes require the same fusion capacity, V-ATPase-dependent acidification, hydrolases, membrane turnover and metabolite-efflux machinery. When mitochondrial cargo occupies this pool, phagosome maturation and antigen processing may be delayed. A high phagocytic burden can reciprocally delay mitophagic completion (93). This molecular competition may provide a plausible route by which organelle-maintenance failure contributes to impaired antimicrobial function, rather than representing two independent lesions.

### 5.4 A self-reinforcing failure of repair

All three states produce incomplete repair. Mitochondrial damage reduces ATP, membrane potential, NAD+ availability, protein import, lysosomal acidification and substrate recycling (50, 94). These abnormalities impede complete removal of damaged material and limit generation of new respiratory capacity (95). Incomplete repair sustains inefficient electron transfer, electron leakage and mtROS production, thereby causing further injury (71). Damage therefore impairs repair, and defective repair allows damage to persist.

Time, cell type and model determine the dominant state. Using mito-QC mice, Sedraoui et al. found that LPS increased general autophagy but did not increase mitolysosomes in limb or diaphragm muscle at 48 h; several mitophagy receptors were unchanged or reduced. This provides direct evidence of inadequate compensatory mitophagy in skeletal muscle and supports an accumulation-dominant interpretation in that model (77). In the immunosuppressive phase, FUNDC1-mediated mitophagy was suppressed in macrophages, and further inhibition worsened metabolic dysfunction, bacterial clearance, lung injury and mortality (96). These observations support insufficient clearance in specific contexts but do not establish a single dominant state across tissues or phases.

### 5.5 What experimental sepsis studies currently support

Taken together, the clearest direct support for an accumulation-dominant interpretation comes from LPS-exposed skeletal muscle, where mito-QC showed no compensatory increase in mitolysosomes despite mitochondrial abnormalities (77). In macrophage and monocyte models, mt-Keima studies document suppressed mitochondrial delivery to acidic compartments (76, 86, 97), but the functional meaning is phase- and model-dependent and does not by itself establish pathological accumulation-dominant uncoupling. During the immunosuppressive phase, impaired FUNDC1-dependent mitophagy worsened macrophage metabolic dysfunction and antibacterial failure (96). Dendritic-cell evidence is consistent with this link: in CLP mice and LPS-treated cells, PINK1 loss impaired PRKN-dependent mitophagy, antigen-presentation markers, cytokine output and T-cell-stimulatory capacity, whereas PRKN activation or DRP1 inhibition partly rescued these *in vitro* defects (98). Nevertheless, intervention results remain context-dependent: increasing mitophagy limited mitochondrial ROS and inflammasome injury in some macrophage models (86, 99), whereas suppressing mitophagy enhanced antibacterial or efferocytic macrophage programs in other acute models (76, 97). Evidence for depletion-dominant uncoupling is more limited and time-dependent: early hepatic mitochondrial loss was followed by biogenesis and recovery rather than persistent unopposed removal (80). Studies that restore autophagic or lysosomal function and improve bacterial killing support lysosomal capacity as functionally limiting (85, 88), but they do not yet prove that mitochondrial cargo occupies a congested lysosomal pool.

Accordingly, no single state can be designated as predominant in sepsis as a whole. Accumulation-dominant uncoupling is most directly supported in the LPS-exposed skeletal-muscle model; selected macrophage studies are consistent with insufficient clearance but do not establish the same state across disease phases. Depletion-dominant and lysosomal-congested states remain testable, tissue- and phase-dependent hypotheses. Definitive assignment requires concurrent measurement of mitochondrial damage, mitophagic delivery and degradation, mitochondrial mass, respiratory function, lysosomal capacity and biogenesis in the same cell population over time.

## 6 From quality-control uncoupling to immune hyporesponsiveness

Mitochondrial injury becomes clinically important when immune cells cannot increase functional output during sustained or renewed stimulation (101). A cell may maintain basal ATP and survive while lacking the energy and metabolic substrates required for host defense. A normal immune-cell count therefore does not necessarily indicate effective immunity (102).

### 6.1 Loss of usable reserve precedes loss of viability

T-cell proliferation, cytokine secretion, immune-synapse formation, granule release, phagocytosis, antigen processing, migration and membrane trafficking all require rapid increases in energy production and biosynthesis (103, 104). Basal respiration reflects resting demand, whereas maximal respiration and spare respiratory capacity quantify the additional output available under stress (105). Quality-control uncoupling reduces this reserve through both impaired performance of retained mitochondria and loss of functional mitochondrial mass.

The deficit first becomes apparent during restimulation. Basal ATP may maintain membrane integrity and housekeeping functions, yet receptor engagement fails to elicit sufficient ATP synthesis, metabolic intermediates, cytoskeletal remodeling or effector-molecule production (106). Specialized immune function may therefore fail before cell viability is lost. Persistent immune hyporesponsiveness reflects not only limited energy availability but also an inability to increase metabolic flux when demand rises (107).

Spare respiratory capacity functions as a cellular stress reserve (108). Basal ATP supports housekeeping processes, whereas spare capacity provides additional output when workload increases. In sepsis, basal metabolism may be preserved despite erosion of this reserve. A resting lymphocyte or monocyte may therefore appear metabolically normal yet fail after receptor engagement, cytokine stimulation or phagocytosis (106). Resting bioenergetic measurements may therefore miss this defect, and clinically stable patients may already have lost immune-cell reserve needed to prevent secondary infection (109).

### 6.2 T cells: inadequate mitochondrial reserve limits renewed activation

Antigen-driven T-cell activation induces glycolysis for rapid growth, but glycolysis alone cannot sustain the complete response (104). T-cell receptor signaling also depends on mitochondrial redox signals and calcium handling (110, 111). Proliferation and effector-molecule production require ATP and biosynthetic intermediates, while sustained effector and memory states depend on mitochondria that can meet repeated demand (103, 112).

Quality-control disruption affects each stage. Damaged mitochondria distort redox and calcium signals, loss of membrane potential and inefficient electron transport reduce respiratory capacity, and impaired substrate utilization together with mitochondrial depletion restricts ATP and biosynthetic intermediates (4). T cells may survive yet fail to divide, secrete interleukin-2 or interferon-γ, exert cytotoxicity or generate durable memory. Cell numbers may therefore be preserved despite inadequate protection against secondary infection (102).

Consistent with this model, splenic CD4+ and CD8+ T cells from CLP mice had lower mitochondrial mass, membrane potential, maximal and spare respiration after activation, and proliferation; patient T cells likewise showed altered activation and exhaustion markers and lower membrane potential (113). Together, these findings support a low-reserve phenotype rather than isolated ATP depletion. Unstable redox and calcium signals impair early activation, while limited biosynthetic intermediates and respiratory reserve constrain expansion, effector output and memory formation (110).

### 6.3 Monocytes and macrophages: failed metabolic transition impairs recall and antigen presentation

Monocytes and macrophages follow a distinct metabolic trajectory. Early glycolysis rapidly supplies ATP for cytokine production and antibacterial activity (114). Oxidative phosphorylation may decrease during this phase, but that decrease alone does not establish mitochondrial injury. Dysfunction emerges when persistent stress prevents an appropriate transition between glycolytic and oxidative programs (115).

Resolution and effective recall require recovery of mitochondrial function, redox balance, lysosomal degradation and metabolic flexibility (116). Quality-control uncoupling disrupts this transition. Damaged mitochondria continue to perturb innate signaling, while lysosomal impairment compromises phagosome maturation and antigen processing (117, 118). HLA-DR expression, ex vivo cytokine responses, phagocytosis and antigen presentation may consequently decline (119). Some scavenging or regulatory functions may persist, yet coordinated antimicrobial recall fails. The central defect is not a uniform shift toward oxidative metabolism, but loss of the flexibility required to adopt the appropriate program for each context (120).

Mitochondrial and lysosomal abnormalities are mechanistically coupled in these cells. Engulfed microbes and damaged mitochondria draw on a shared pool of trafficking, lysosomal fusion, V-ATPase-dependent acidification, hydrolase activity, membrane recycling and substrate-efflux capacity. If mitochondrial cargo saturates this pool, mitophagic completion slows while phagosome maturation, microbial killing and antigen processing compete for the same terminal machinery. Sustained phagocytic load can reciprocally delay mitochondrial clearance. mtROS and mtDNA may sustain selected innate signaling activity (121), whereas limited processing and energy reserve reduce microbial killing, peptide loading, co-stimulation and HLA-DR-dependent antigen presentation (122). Taken together, persistent innate signaling and defective antimicrobial processing support a model in which organelle-maintenance failure contributes to monocyte hyporesponsiveness (123).

### 6.4 Why inflammation and immune hyporesponsiveness coexist

Damage accumulation and reserve depletion can drive opposing immune outputs. Damaged mitochondria sustain mtROS and danger signaling (7), but reduced mitochondrial reserve weakens antigen presentation, proliferation, phagocytosis and cytotoxicity (123). A cell may therefore retain inflammatory activity while performing antimicrobial functions less effectively.

Inflammation and immune hyporesponsiveness are not necessarily consecutive or mutually exclusive states (2). Mitochondrial signals can still activate innate sensors when adaptive expansion and monocyte recall are impaired (124). At the tissue level, inflamed areas can exist together with profoundly hyporesponsive circulating cells (119). Distinguishing danger signaling from specialized immune functions clarifies this apparent contradiction.

## 7 The reverse feedback that stabilizes the mitochondrial–immune trap

Quality-control uncoupling can initiate immune dysfunction, while inhibitory immune programs may stabilize persistent hyporesponsiveness by constraining cellular metabolism (125). This reverse feedback may explain why immune function remains depressed after the initial inflammatory stimulus subsides.

Persistent antigen stimulation, regulatory cytokines, altered metabolite pools and sustained stress can maintain PD-1 and related inhibitory pathways (126). Transient expression during acute activation is insufficient to define exhaustion, which instead combines persistent inhibitory signaling, impaired recall and limited metabolic reserve. These mechanisms can suppress PI3K–AKT–mTOR activity, reduce glucose-transporter induction and anabolic metabolism, and restrict programs that support functional mitochondrial replacement (127, 128).

This feedback occurs when cells most require substrates and biosynthesis to repair their mitochondrial network. Reduced glucose uptake and anabolism impair NAD+ regeneration, antioxidant capacity, membrane synthesis, respiratory-protein synthesis and mitochondrial biogenesis (129). Functional replacement becomes less effective, respiratory capacity declines and immune dysfunction worsens (130).

The forward and reverse arms operate on different time scales but converge on the same limiting resource. The forward arm reflects physical loss or dysfunction of mitochondrial capacity that becomes evident as immune workload increases. The reverse arm reflects persistent inhibitory signals that suppress the substrate uptake and biosynthesis required to restore that capacity (126). When both arms are active, improved perfusion or pathogen control may relieve the initial injury while respiratory reserve remains low and metabolism remains constrained (3). Recovery therefore requires both reduced damage and renewed access to pathways that complete clearance and replacement.

Persistent metabolic disturbance may further hinder the reversal of immune hyporesponsiveness by affecting chromatin regulation through metabolite-sensitive pathways (131, 132). These changes may stabilize low-response programs, but their precise connection to mitochondrial quality-control failure in human sepsis remains unclear (133).

The resulting circuit is bidirectional. Mitochondrial quality-control uncoupling removes reserve needed for immune function, while inhibitory signals restrict the metabolic and biosynthetic programs required for functional replacement. The injured system lacks capacity for recovery, and the inhibitory state prevents that capacity from being rebuilt. This circuit may explain why control of the primary infection can precede restoration of immune competence. The initial insult may resolve while mitochondrial reserve remains depleted and metabolic replacement remains constrained (124).

## 8 Translational implications: identify the dominant failure before intervening

A coordination-centered framework changes how biomarkers and treatments are interpreted because no single measurement of mtDNA, LC3, PINK1, mitochondrial mass or PD-1 identifies the dominant state (134, 135). We define state-matched longitudinal phenotyping as repeated, cell-type-specific assessment of damage burden, completed flux, residual reserve, cell composition and disease phase (102). Each time point should integrate ongoing mitochondrial damage, functional and recovery capacity and residual immune function (2). A prospective study of 37 septic ICU patients illustrated this rationale: PBMC mtDNA copy number was reduced and plasma D-loop DNA was increased on day 1, whereas biogenesis-associated transcription increased and mtDNA copy number recovered by day 5; early activation of mitochondrial biogenesis was associated with ICU discharge within one week (136). Although the study did not measure mitophagic flux or resolve PBMC subsets, the divergence between damage and recovery markers shows why state assignment requires repeated, multidimensional measurements rather than a single baseline value.

### 8.1 Distinguishing accumulation, depletion, and lysosomal congestion

Markers such as cell-free mtDNA, mtDNA damage, membrane-potential loss and oxidized mitochondrial lipids can indicate damage burden (7, 137). They reflect stress or release but cannot distinguish active injury from blocked clearance, cell death or recovery (138). Interpretation therefore requires concurrent assessment of mitochondrial mass and function (139).

Basal and maximal respiration, ATP-linked respiration, spare respiratory capacity, membrane potential, mitochondrial mass and redox state characterize functional capacity (140). Respiration measured without mitochondrial mass may overestimate efficiency when only a small number of active organelles remain; mitochondrial mass measured without maximal function may miss a large but inefficient network. Cell-type-specific assays can also reduce confounding from changes in peripheral blood mononuclear-cell composition (141).

Direct functional tests define the immune work that cells can still perform. Monocyte HLA-DR, ex vivo cytokine production, phagocytic capacity, T-cell proliferation and cytokine production, and natural killer-cell degranulation provide complementary information (142, 143). Mitochondrial damage may coexist with preserved immune capacity, while lower damage markers may reflect prior mitochondrial depletion. Functional assays link organelle state to immune consequences.

### 8.2 State-matched mechanistic priorities

These priorities combine experimental observations with model-derived predictions and should not be treated as established treatments. Current direct evidence most strongly supports insufficient clearance in specific skeletal-muscle and myeloid-cell models rather than a universal state across sepsis (76, 77, 96). In accumulation-dominant uncoupling, the priority is to reduce ongoing injury and support selective, completed clearance. In a lysosomal-constrained state, further stimulation of pathway entry should be considered only after fusion, acidification, degradation and substrate export have been assessed (144).

Direct evidence that excessive mitochondrial clearance causes depletion-dominant uncoupling in sepsis remains insufficient. Experimental enhancement of PINK1-dependent mitophagy protected LPS-stimulated macrophages and CLP lungs in one intervention study (145), while coordinated increases in mitophagy and biogenesis accompanied improved mitochondrial integrity and survival in septic lung models (146). Depletion-dominant uncoupling should therefore be tested with paired measurements of flux, mitochondrial mass and replacement rather than inferred from fragmentation or low mitochondrial mass alone.

The model further predicts that successful immune reactivation depends on residual metabolic capacity. IL-7, GM-CSF, interferon-γ or checkpoint-directed strategies may produce more durable effects in viable transitional cells that retain respiratory capacity and metabolic plasticity (147, 148). Relieving inhibitory constraints in severely exhausted cells may produce only transient activation, whereas increasing mitochondrial capacity without overcoming persistent inhibition may leave immune function restricted (149). Therefore, an effective recovery requires both mitochondrial competence and immune responsiveness. This state-matched framework is illustrated in **Figure 2**.

**Figure 2:**
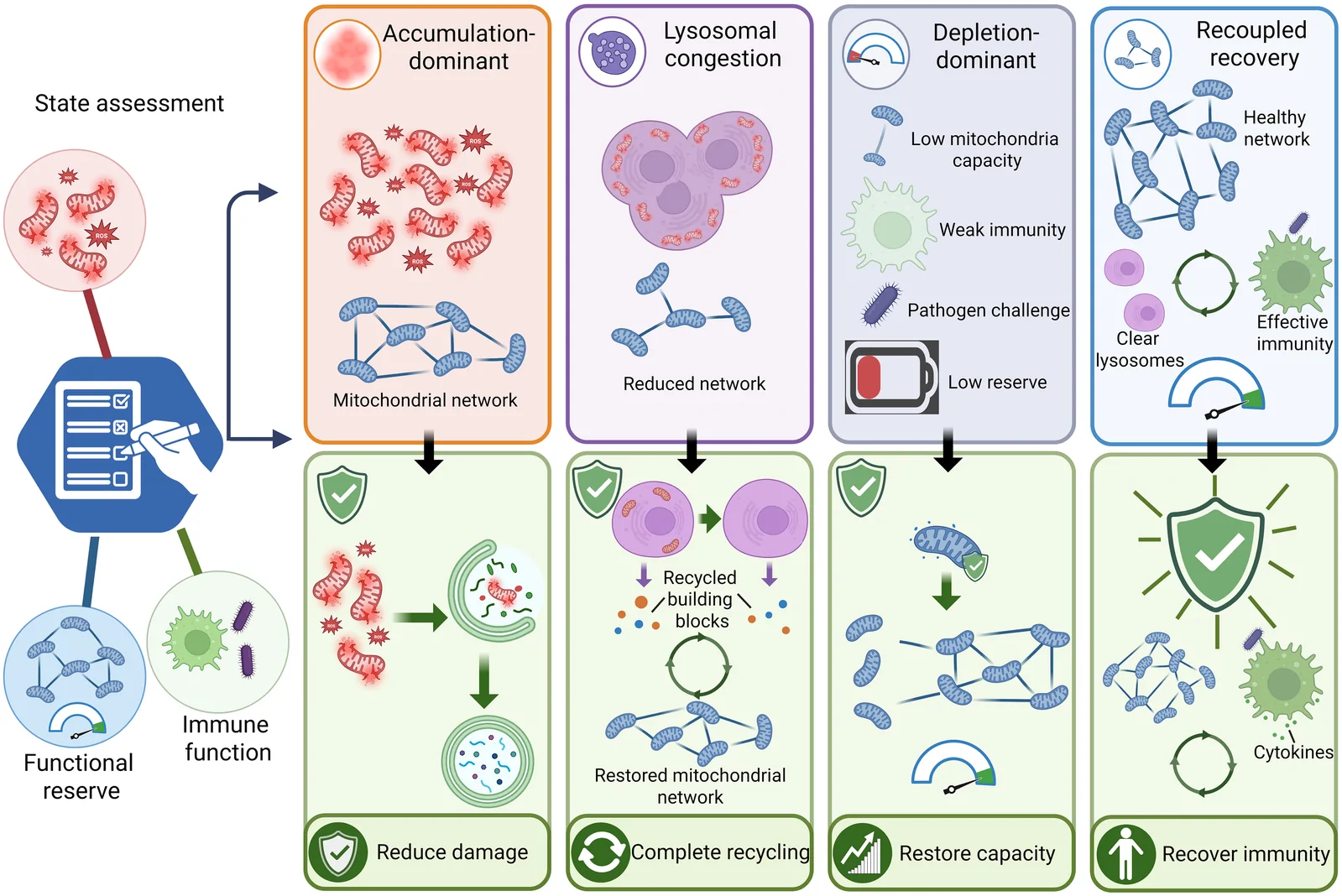
State-based framework for mitochondrial dysfunction and recovery in sepsis. Three dimensions—damage burden, functional reserve, and immune function—are used to identify the dominant mitochondrial state. Accumulation-dominant dysfunction reflects persistent damaged mitochondria, lysosomal congestion reflects incomplete degradation and recycling, and depletion-dominant dysfunction reflects loss of mitochondrial capacity and respiratory reserve. Each state is linked to a corresponding recovery strategy: reducing damage, completing recycling, or restoring capacity. Successful correction of these defects may lead to recoupled recovery, with restoration of mitochondrial function and immune responsiveness. Created in BioRender. Zhang, Y. (2026) https://BioRender.com/rdr3wp3 (The abbreviations are given in the Abbreviations part).

## 9 Testable predictions and unresolved questions

The quality-control uncoupling model yields three testable predictions. First, the relationship between mitochondrial damage and completed recovery should explain immune function better than any single pathway-entry marker. Mitophagy initiation should be beneficial only when lysosomal completion and functional replacement remain sufficient (22).

The model also predicts a temporal sequence. Mitochondrial injury and pathway-entry signals should precede failure of lysosomal completion or functional replacement; loss of residual respiratory capacity and immune function should then become most apparent during restimulation (13, 150). An intervention that increases an upstream marker without restoring completed flux, respiratory reserve and recall function would not support the proposed causal chain.

Accordingly, the same clearance-directed intervention may have opposite effects across states. It may improve function when damaged organelles accumulate in cells with active lysosomes and sufficient replacement capacity, but worsen function after mitochondrial mass and respiratory reserve are depleted (12).

Third, residual respiratory reserve should predict sustained responses to immune stimulation (100). Cell-type-specific measurements in monocytes and T cells should outperform aggregate values from heterogeneous peripheral blood mononuclear cells because these cells differ in metabolic programs, lysosomal demands and dependence on mitochondrial reserves (139, 141).

Several mechanistic questions remain unresolved. The relative contributions of PRKN-dependent and receptor-mediated mitophagy may differ among immune-cell subsets and disease phases (29). Direct studies should determine whether fragmentation is followed by selective mitophagic commitment, whether mitochondrial removal outpaces biogenesis, and whether mitochondrial cargo stalls within lysosomes in immune cells. Finally, causal studies should determine whether recoupling clearance, recycling and functional replacement reverses immune hyporesponsiveness or merely improves mitochondrial markers.

## 10 Discussion and conclusions

Sepsis-associated mitochondrial injury and persistent immune hyporesponsiveness are often treated as separate problems. We propose that mitochondrial quality-control uncoupling links them by disrupting coordination among damage containment, selective removal, lysosomal degradation and recycling, and functional replacement.

Direct reporter and functional-perturbation studies support insufficient compensatory mitophagy in LPS-exposed skeletal muscle and selected macrophage states (76, 77, 86, 96, 97, 99), but they do not establish a single state across all tissues or phases. Evidence for persistent removal exceeding replacement and for mitochondrial-cargo-specific lysosomal congestion is less direct (80, 85, 88). We therefore present the three states as experimentally testable, cell- and phase-specific configurations rather than established universal endotypes.

When damage exceeds completed clearance, dysfunctional mitochondria accumulate and sustain redox and danger signaling (151). When removal exceeds replacement, mitochondrial mass and respiratory capacity decline (11). When lysosomal degradation fails, mitochondria leave the functional network before cargo is fully processed, producing simultaneous depletion and retained debris (27). These states may occur in different immune cells or disease phases, but they converge on the same outcome: viable cells cannot increase metabolic and biosynthetic output during renewed challenge (105). Inhibitory immune signals can then restrict substrate utilization and functional mitochondrial replacement, reinforcing the mitochondrial–immune trap.

This framework explains how inflammation can persist as antimicrobial immunity declines (2), why incomplete clearance can coexist with increased mitophagy markers (18), and how accumulation and depletion can reflect distinct failures within one maintenance system. Interventions should therefore target the dominant defect rather than uniformly increasing mitophagy or immune activation. Longitudinal, cell-type-specific studies are needed to test whether restoring coordination improves both mitochondrial repair and immune function (102).
